# Supplementary material for: Application of a Nomogram Model in Predicting Postoperative Delirium Following Percutaneous Coronary Intervention
Source: Bioengineering (Basel). 2025 Jun 11;12(6):637. doi: 10.3390/bioengineering12060637 (PMC12189321; doi:10.3390/bioengineering12060637)

Tabel S1:Missing number (%) for Characteristics

Characteristic	Missing number (%)
Gender	0(0)
Age	0(0)
Admission Type	0(0)
Hb	42(13.4)
PLT	42(13.4)
WBC	45(14.3)
BUN	35(11.1)
Cr	36(11.5)
INR	61(19.4)
APTT	53(16.9)
Bicarbonate	36(11.5)
Serum Potassium	33(10.5)
Serum Sodium	33(10.5)
Blood Glucose	39(12.4)
AG	36(11.5)
MBP	7(2.2)
HR	4(1.2)
SP02	6(1.9)
Benzodiazepine Use	0(0)
Vasoactive Drug Therapy	0(0)

Table S2: Descriptive statistical analysis of data before and after imputation

Characteristic	**Before Imputation Data**, N = 313	**After Imputation Data**, N = 313	p-value
Gender			>0.999
Male	186 (59%)	186 (59%)	
Female	127 (41%)	127 (41%)	
Age	71.00 (62.00, 80.00)	71.00 (62.00, 80.00)	>0.999
Admission Type			>0.999
Emergency	149 (48%)	149 (48%)	
Urgent	128 (41%)	128 (41%)	
Other	36 (12%)	36 (12%)	
Hb	11.70 (10.00, 13.30)	11.70 (10.00, 13.30)	0.873
PLT	208.00 (157.50, 275.50)	207.00 (154.00, 275.00)	0.800
WBC	10.65 (7.58, 14.73)	10.20 (7.40, 14.50)	0.448
BUN	23.00 (17.00, 33.00)	24.00 (17.00, 34.00)	0.845
Cr	1.10 (0.80, 1.50)	1.10 (0.80, 1.50)	0.772
INR	1.20 (1.10, 1.40)	1.20 (1.10, 1.40)	0.690
APTT	37.55 (29.60, 62.35)	35.90 (29.40, 60.00)	0.617
Bicarbonate	24.00 (21.00, 26.00)	24.00 (21.00, 26.00)	0.779
Serum Potassium	4.10 (3.80, 4.70)	4.10 (3.80, 4.70)	0.993
Serum Sodium	138.00 (135.00, 140.00)	138.00 (135.00, 140.00)	0.847
Blood Glucose	136.50 (107.00, 187.25)	139.00 (107.00, 185.00)	0.864
AG	15.00 (13.00, 18.00)	15.00 (13.00, 18.00)	0.672
MBP	81.00 (71.00, 93.00)	81.00 (70.00, 93.00)	0.959
HR	88.00 (72.00, 104.00)	88.00 (71.00, 104.00)	0.994
SP02	97.00 (94.00, 99.00)	97.00 (94.00, 99.00)	0.957
Benzodiazepine Use	119 (38%)	119 (38%)	>0.999
Vasoactive Drug Therapy	89 (28%)	89 (28%)	>0.999

Table S3:Baseline Comparison of Patients in Training and Test Sets

**Characteristic**	**Train**, N = 219	**Test**, N = 94	**p-value**
Gender			0.399
Male	134 (61%)	52 (55%)	
Female	85 (39%)	42 (45%)	
Age	71.00 (62.00, 79.00)	73.00 (62.25, 83.00)	0.134
Admission Type			0.286
Emergency	100 (46%)	49 (52%)	
Urgent	90 (41%)	38 (40%)	
Other	29 (13%)	7 (7.4%)	
Hb	11.60 (9.85, 13.35)	11.80 (10.25, 13.18)	0.585
PLT	208.00 (153.00, 274.50)	203.50 (164.75, 274.00)	0.494
WBC	10.30 (7.40, 14.50)	10.20 (7.33, 14.45)	0.852
BUN	23.00 (16.50, 33.00)	25.00 (17.00, 35.00)	0.297
Cr	1.10 (0.80, 1.50)	1.20 (0.83, 1.60)	0.291
INR	1.20 (1.10, 1.40)	1.20 (1.10, 1.40)	0.225
APTT	37.50 (29.65, 64.05)	34.80 (28.00, 52.80)	0.103
Bicarbonate	23.00 (21.00, 26.00)	24.00 (21.00, 27.00)	0.442
Serum Potassium	4.10 (3.80, 4.70)	4.10 (3.80, 4.70)	0.958
Serum Sodium	138.00 (135.00, 140.00)	139.00 (136.00, 141.00)	0.367
Blood Glucose	137.00 (107.00, 183.00)	142.00 (113.50, 190.25)	0.458
AG	15.00 (13.00, 18.00)	15.00 (13.00, 18.00)	0.845
MBP	81.00 (71.00, 94.00)	79.50 (67.00, 93.00)	0.467
HR	87.00 (71.00, 103.00)	89.00 (75.00, 105.00)	0.267
SP02	97.00 (95.00, 100.00)	96.00 (94.00, 98.00)	0.094
Benzodiazepine Use	81 (37%)	38 (40%)	0.654
Vasoactive Drug Therapy	58 (26%)	31 (33%)	0.303
Hospital Mortality	19 (8.7%)	6 (6.4%)	0.647
Hospital LOS	10.00 (5.00, 15.50)	8.50 (5.00, 15.00)	0.793
Delirium	55 (25%)	24 (26%)	>0.999

Figure S1: Comparison of Data Distribution Before and After Imputation

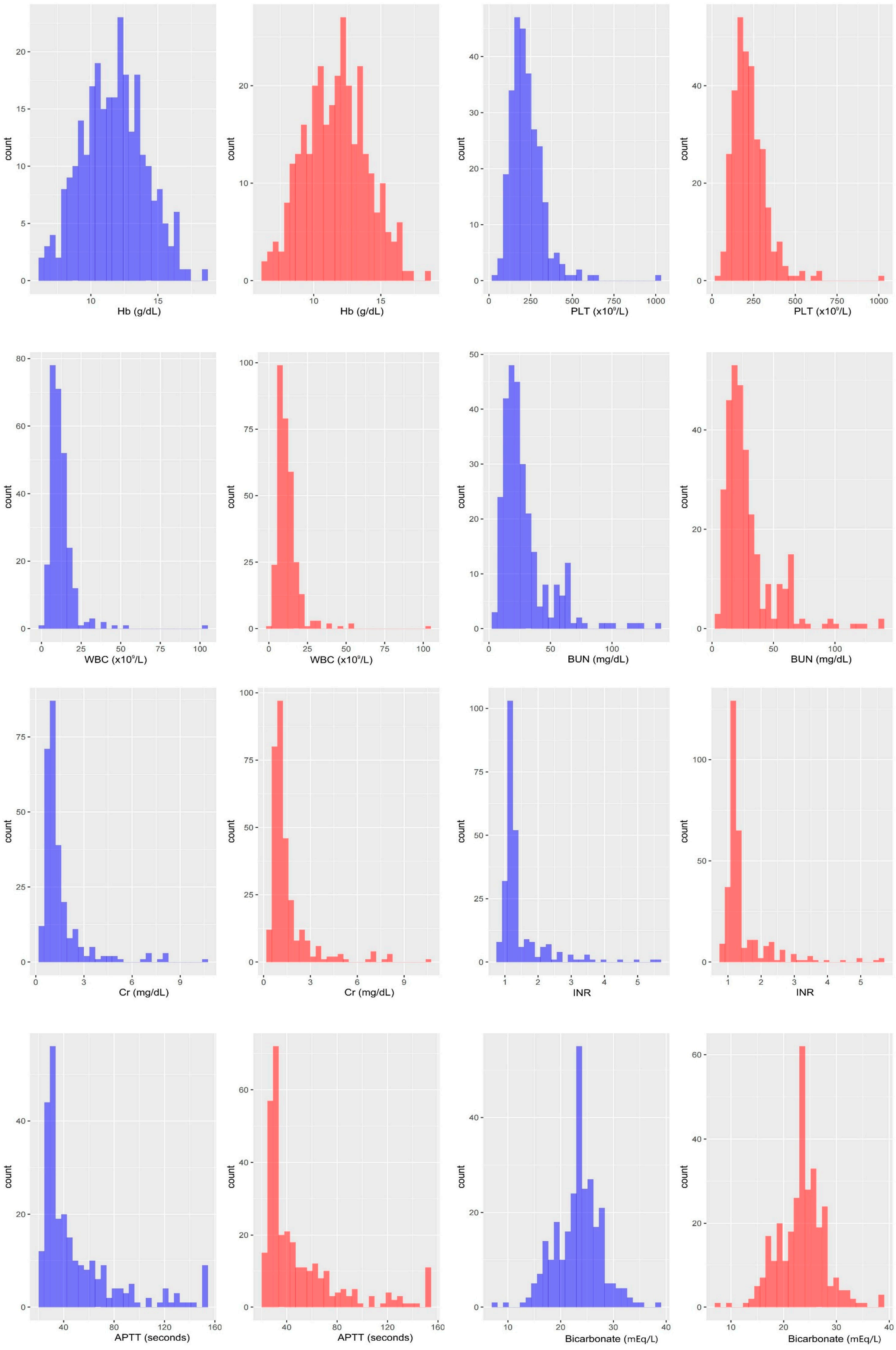

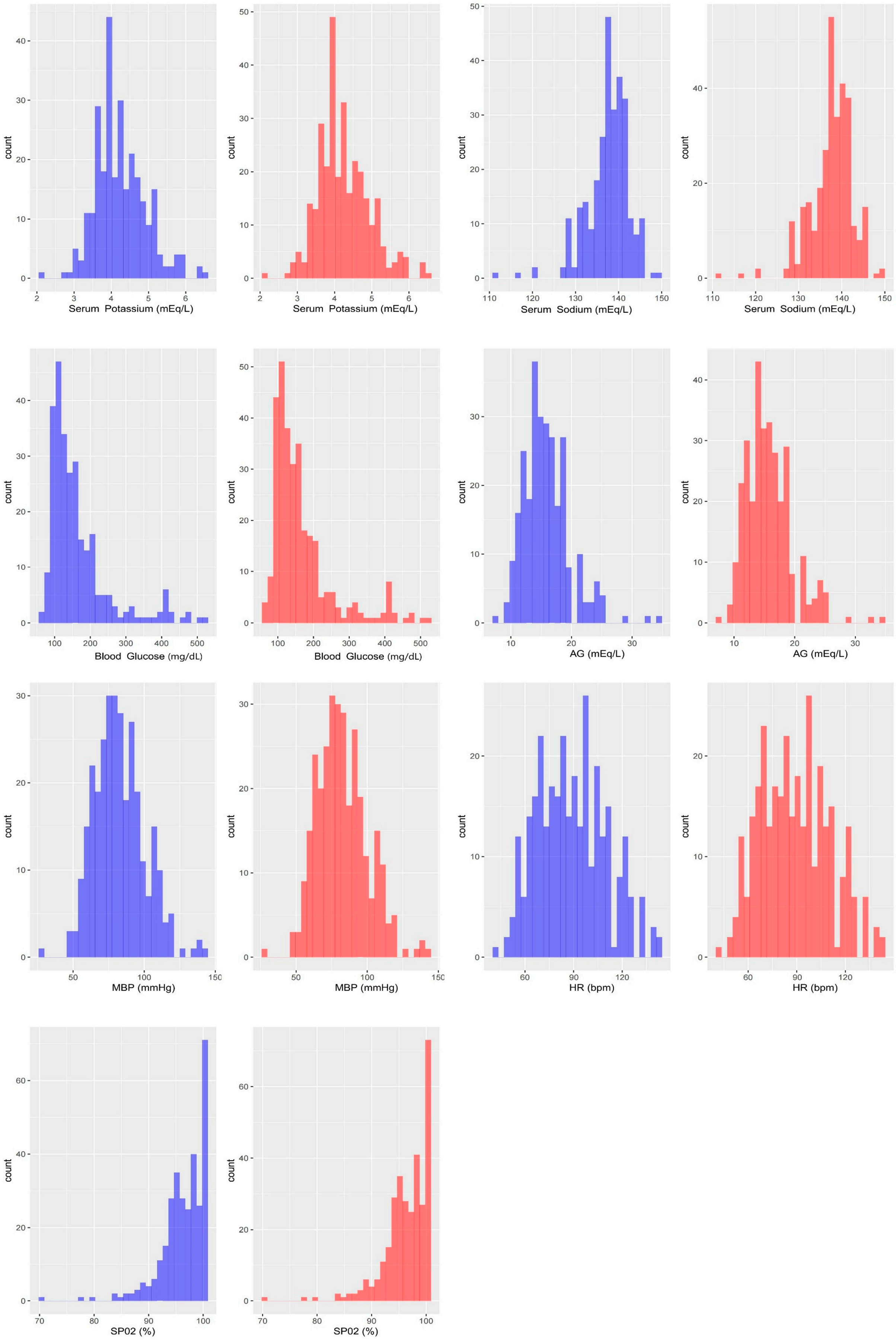

Blue: data before imputation; Red: data after imputation

Figure S2: Bootstrap AUC Distribution

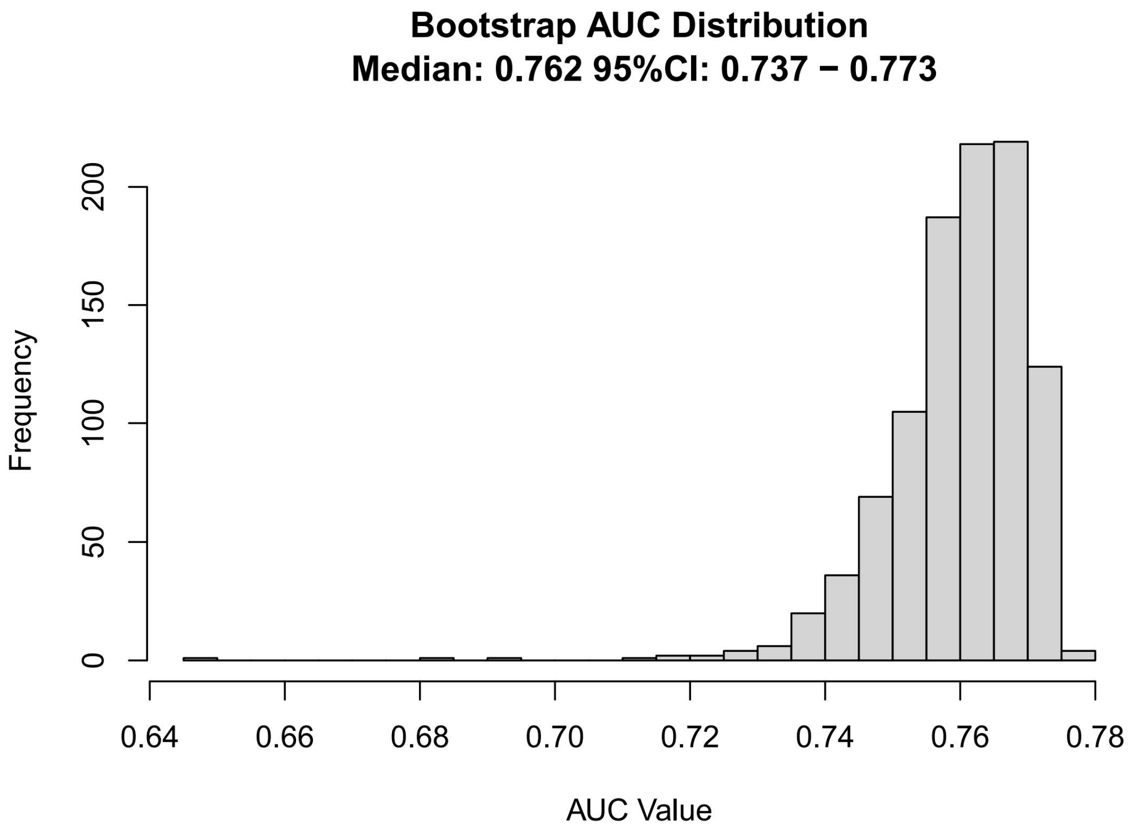

Supplement: Supplementary file 1 [file bioengineering-12-00637-s001.zip › bioengineering-3662567-supplementary.pdf]
